# Imaging of *Pseudomonas aeruginosa* infection with Ga-68 labelled pyoverdine for positron emission tomography

**DOI:** 10.1038/s41598-018-33895-w

**Published:** 2018-10-24

**Authors:** Milos Petrik, Eva Umlaufova, Vladislav Raclavsky, Andrea Palyzova, Vladimir Havlicek, Hubertus Haas, Zbynek Novy, Dalibor Dolezal, Marian Hajduch, Clemens Decristoforo

**Affiliations:** 1 Institute of Molecular and Translational Medicine, Faculty of Medicine and Dentistry, Palacky University, Olomouc, Czech Republic; 2 Department of Microbiology, Faculty of Medicine and Dentistry, Palacky University, Olomouc, Czech Republic; 3 Institute of Microbiology of the Czech Academy of Sciences, v.v.i, Prague, Czech Republic; 4 Regional Centre of Advanced Technologies and Materials, Department of Analytical Chemistry, Faculty of Science, Palacky University, Olomouc, Czech Republic; 5 Division of Molecular Biology/Biocenter, Medical University Innsbruck, Innsbruck, Austria; 6 Clinical Department of Nuclear Medicine, Medical University Innsbruck, Innsbruck, Austria

## Abstract

*Pseudomonas aeruginosa* is an increasingly prevalent opportunistic pathogen that causes a variety of life-threatening nosocomial infections. Novel strategies for the development of new antibacterial treatments as well as diagnostic tools are needed. One of the novel diagnostic strategies for the detection of infection could be the utilization of siderophores. Siderophores are low-molecular-weight chelators produced by microbes to scavenge essential iron. Replacing iron in siderophores by suitable radiometals, such as Ga-68 for positron emission tomography (PET) imaging, opens approaches for targeted imaging of infection. Here we report on pyoverdine PAO1 (PVD-PAO1), a siderophore produced by *P. aeruginosa*, labelled with Ga-68 for specific imaging of *Pseudomonas* infections. PVD-PAO1 was labelled with Ga-68 with high radiochemical purity. The resulting complex showed hydrophilic properties, low protein binding and high stability in human serum. *In vitro* uptake of ^68^Ga-PVD-PAO1 was highly dependent on the type of microbial culture. In normal mice ^68^Ga-PVD-PAO1 showed rapid pharmacokinetics with urinary excretion. PET imaging in infected animals displayed specific accumulation of ^68^Ga-PVD-PAO1 in infected tissues and better distribution than clinically used ^18^F-fluorodeoxyglucose (^18^F-FDG) and ^68^Ga-citrate. Ga-68 labelled pyoverdine PAO1 seems to be a promising agent for imaging of *P. aeruginosa* infections by means of PET.

## Introduction

Invasive microbial infections are one of the leading causes of morbidity and mortality especially in immunocompromised hosts^1,2^. Early and accurate identification of a causative microorganism in such patients is crucial for their survival. However, current diagnostic methods including culture, serology, molecular and radiology techniques used for pathogen identification are often slow, invasive, lack sensitivity and/or specificity, and are unable to localize infection in the body. The availability of rapid and reliable diagnostic tool for infectious diseases represents a major unmet need in managing critically ill patients^2^.

The lung is the most frequently involved organ in microbial infections in the immunodeficient host. One of the most common pathogens causing life-threatening pneumonia is *Pseudomonas aeruginosa*, an easily adaptable Gram-negative bacterium colonizing and/or invading human hosts and causing wide range of nosocomial infections^3^. The main risk factors for the development of such infections include neutropenia, cystic fibrosis, severe burns and foreign device installations^4^. Rapid and reliable diagnosis of pneumonia caused by *P. aeruginosa* is complicated^5^. Standard methods for assessing respiratory infections with *P. aeruginosa* are suboptimal and new diagnostic tools to identify the causative pathogen are needed^3,6^. One of the new diagnostic strategies for the specific detection of manifested infection could be the utilization of labelled siderophores.

Siderophores are low-molecular mass iron chelators produced by nearly all microorganisms and some plants for iron acquisition and storage^7,8^. Iron is an essential metal for a plethora of cellular events and therefore an indispensable micronutrient for almost all living organisms including microbes^9^. During infection, pathogens encounter an essentially iron-free environment as the available iron is tightly sequestered by host proteins, e.g., haemoglobin, transferrin, lactoferrin, and ferritin^10^. Consequently, pathogens had to evolve mechanisms for “stealing” host iron. *P. aeruginosa* can use different strategies to acquire iron including production of siderophores, uptake of exogenous siderophores (xenosiderophores) produced by other microorganisms, uptake of heme molecules from the host or the use of Feo system^11^.

*P. aeruginosa* produces different types of siderophores including pyoverdine (PVD) and uses highly efficient FpvA transporters specific for ferripyoverdine uptake^12,13^. PVD is involved in nutrition, biofilm control, cell-to-cell communication and virulence regulation, thus playing an important role in the pathophysiology of *P. aeruginosa*^14^. Our purpose was the preclinical evaluation of PVD radiolabelled with Ga-68 for imaging of *P. aeruginosa* infection using positron emission tomography (PET). Ga-68 is a positron emitter with comparable physicochemical properties to those of iron (III) and binds with high affinity to various siderophores^15^. A similar Trojan horse strategy utilizing different Ga-68 labelled siderophores was successfully established for imaging of fungal pathogen *Aspergillus fumigatus* in the past^16,17^.

## Results

### Radiolabelling and *in vitro* characterization

**Figure 1:**
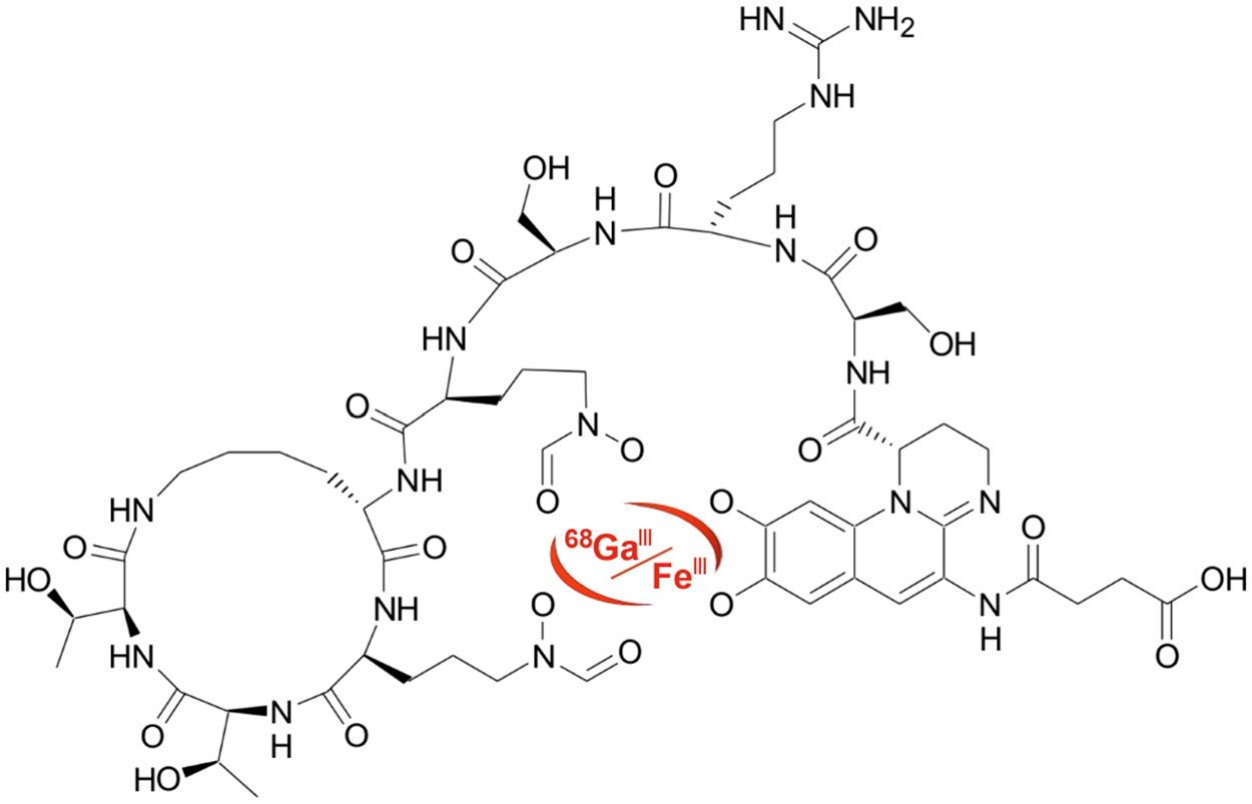
The chemical structure of Fe/^68^Ga-Pyoverdine PAO1 (PVD-PAO1).

Pyoverdine PAO1 (PVD-PAO1) (Fig. 1) was labelled with Ga-68 with high radiochemical purity (>95%). The resulting complex showed hydrophilic properties (log P = −3.07 ± 0.08) with low values of protein binding even 120 min after incubation in human serum (<3%). ^68^Ga-PVD-PAO1 displayed excellent stability in human serum (~95%) as well as in DTPA solution (~90%) and rapid *in vitro* instability in highly concentrated FeCl_3_ solution. A summary of the analytical data and *in vitro* characteristics of ^68^Ga-PVD-PAO1 is given in Supplementary Fig. S1 and Supplementary Table S1.

### *In vitro* uptake of ^68^Ga-PVD-PAO1 by microbial cultures

**Figure 2:**
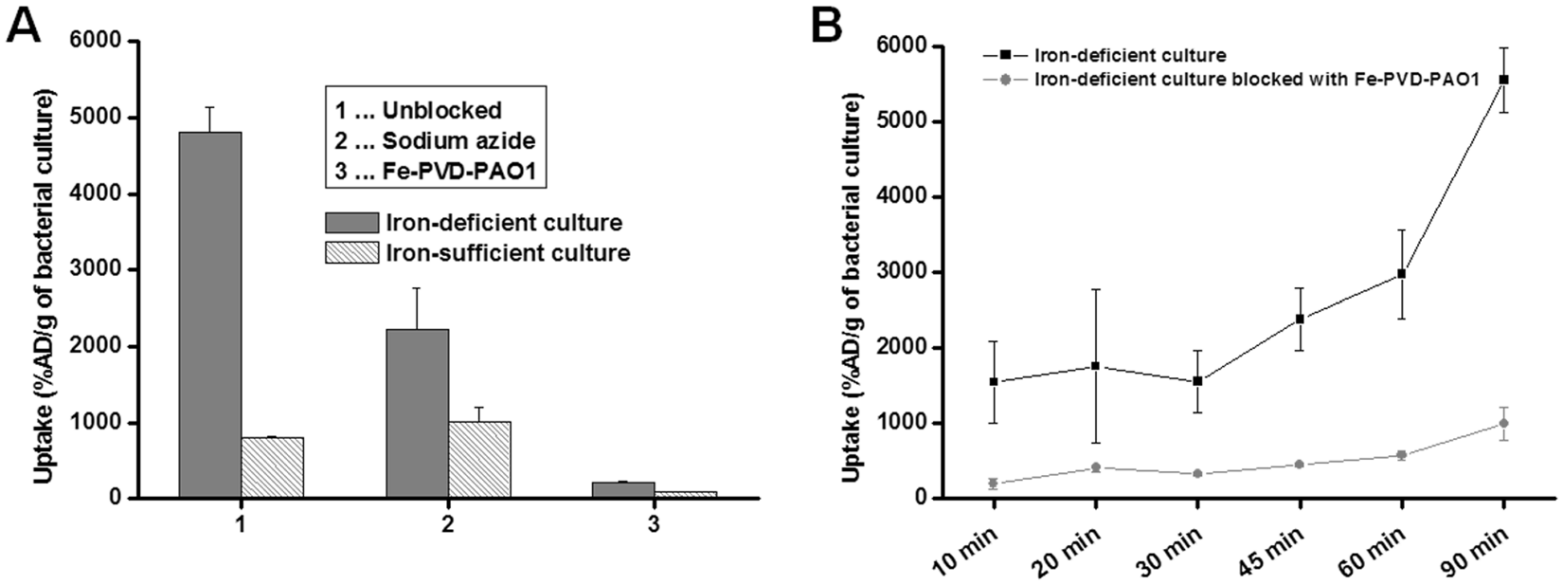
(**A**) *In vitro* uptake of ^68^Ga-PVD-PAO1 in *P. aeruginosa* after 45 min of incubation under varying conditions (2 mM sodium azide and excess of Fe-PVD-PAO1) in iron-deficient and iron-sufficient conditions. (**B**) *In vitro* uptake of ^68^Ga-PVD-PAO1 in *P. aeruginosa* over time under iron-deficient conditions and in the presence of excess of Fe-PVD-PAO1.

**Figure 3:**
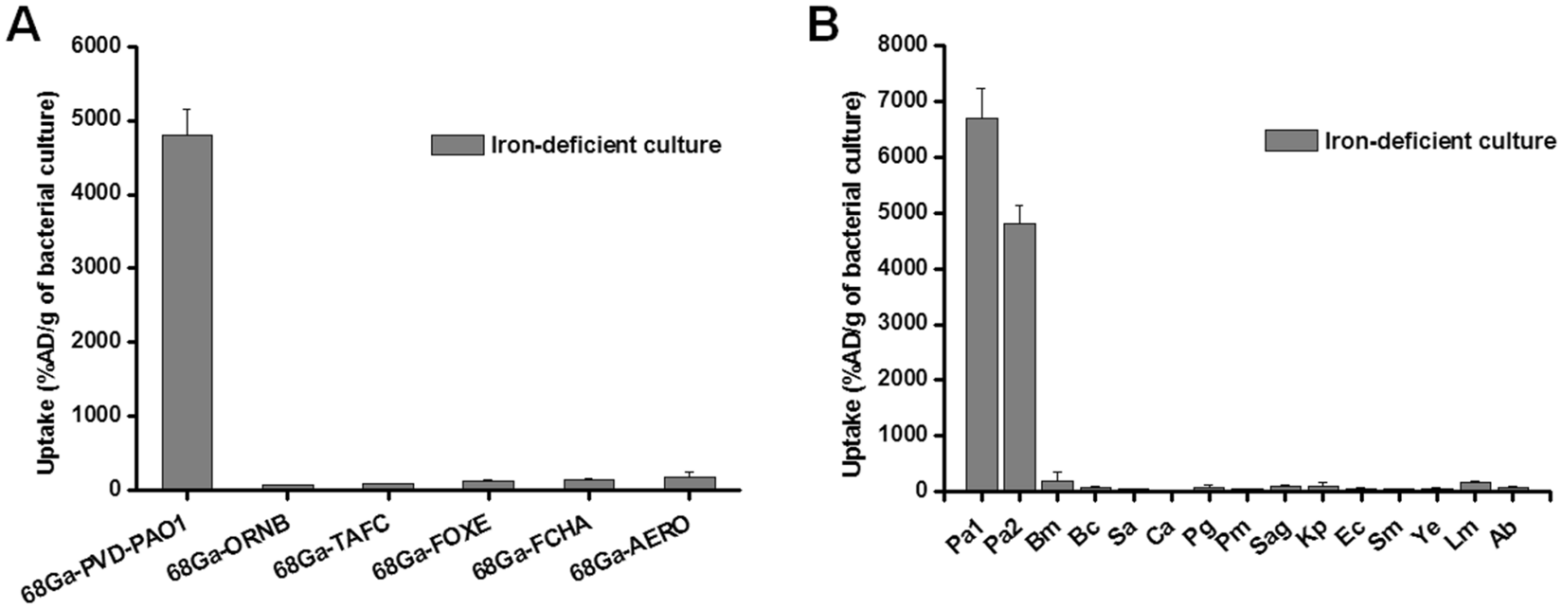
(**A**) *In vitro* uptake of different Ga-68 labelled siderophores in *P. aeruginosa* after 45 min of incubation under iron-deficient conditions. (**B**) *In vitro* uptake of ^68^Ga-PVD-PAO1 in various microbial cultures (Pa1 = *Pseudomonas aeruginosa* ATCC 15692, Pa2 = *Pseudomonas aeruginosa* 1019, Bm = *Burkholderia multivorans* 1926, Bc = *Burkholderia cenocepacia* 2029, Sa = *Staphylococcus aureus* 230, Ca = *Candida albicans* 1265, Pg = *Pseudomonas grimontii* 754, Pm = *Pseudomonas monteilii* 2260, Sag = S*treptococcus agalactiae* 487, Kp = *Klebsiella pneumoniae* 2102, Ec = *Escherichia coli* 2463, Sm = *Stenotrophomonas maltophilia* 303, Ye = *Yersinia enterocolitica* 2871, Lm = *Listeria monocytogenes*, Ab = *Acinetobacter baumannii* 2377) after 45 min of incubation under iron-deficient conditions.

Uptake of ^68^Ga-PVD-PAO1 by *P. aeruginosa* was highly dependent on the bacterial culture conditions. Iron-deficient but not iron-sufficient cultures displayed high uptake that could be blocked with an excess (10 µM) of cold iron PVD-PAO1 and partly with NaN_3_, indicating a specific and energy-dependent uptake mechanism (Fig. 2A). Uptake was observed up to 90 min after incubation without saturation, again sensitive to blocking with Fe-PVD-PAO1 (Fig. 2B). Uptake study of different siderophores not produced by *P. aeruginosa* radiolabelled with Ga-68 in iron-deficient *P. aeruginosa* cultures showed negligible uptake of these compounds compared to ^68^Ga-PVD-PAO1 (Fig. 3A). Figure 3B summarizes the uptake of ^68^Ga-PVD-PAO1 in different microorganisms. Under iron-deficient conditions ^68^Ga-PVD-PAO1 showed much higher uptake by *P. aeruginosa* compared to other tested microbial cultures in which minor or no uptake was observed, indicating high specificity of ^68^Ga-PVD-PAO1 for *P. aeruginosa*.

### *Ex vivo* biodistribution in mice and rats

**Figure 4:**
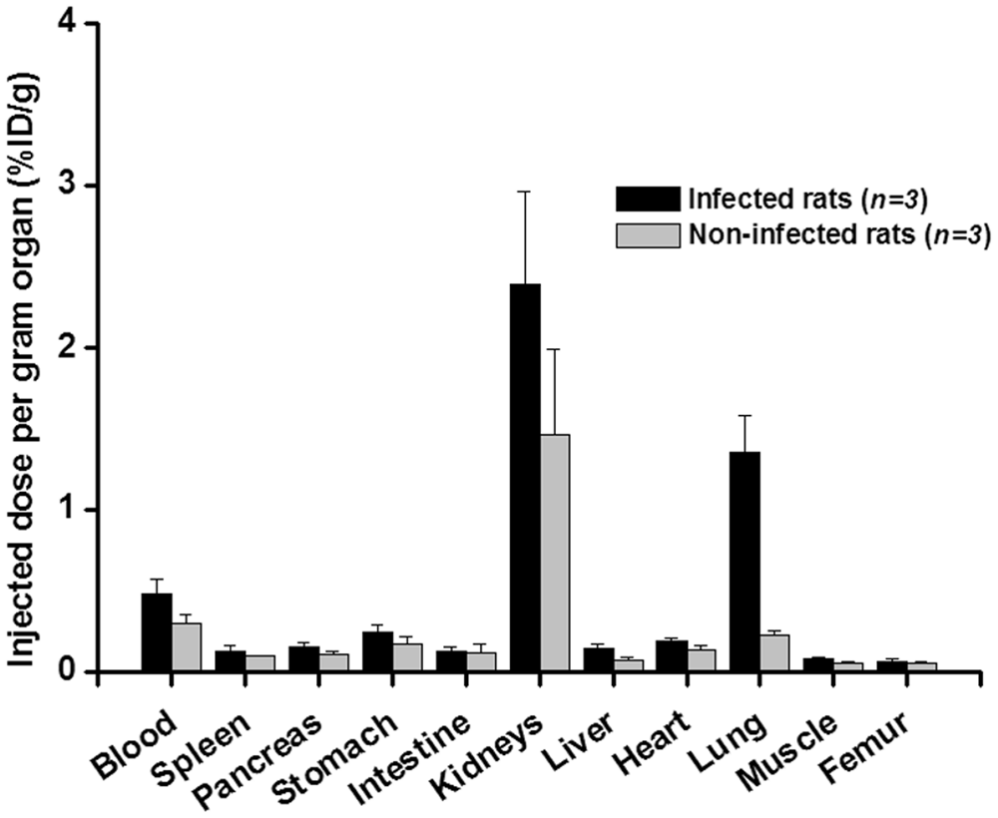
Biodistribution of ^68^Ga-PVD-PAO1 in intratracheally infected and non-infected Lewis rats 45 min after injection.

In non-infected Balb/c mice (Supplementary Fig. S2A), ^68^Ga-PVD-PAO1 displayed rapid excretion via the renal system and showed minimal retention in blood and other organs, even at short times (30 and 90 min) after injection. Biodistribution of ^68^Ga-PVD-PAO1 in intratracheally infected and non-infected Lewis rats 45 min p.i. (Fig. 4) did not show any significant difference in organ uptake, except for lung uptake (1.35 ± 0.23%ID/g in infected rats; 0.23 ± 0.03%ID/g in non-infected rats; P < 0.005).

### PET/CT imaging in mice and rats

**Figure 5:**
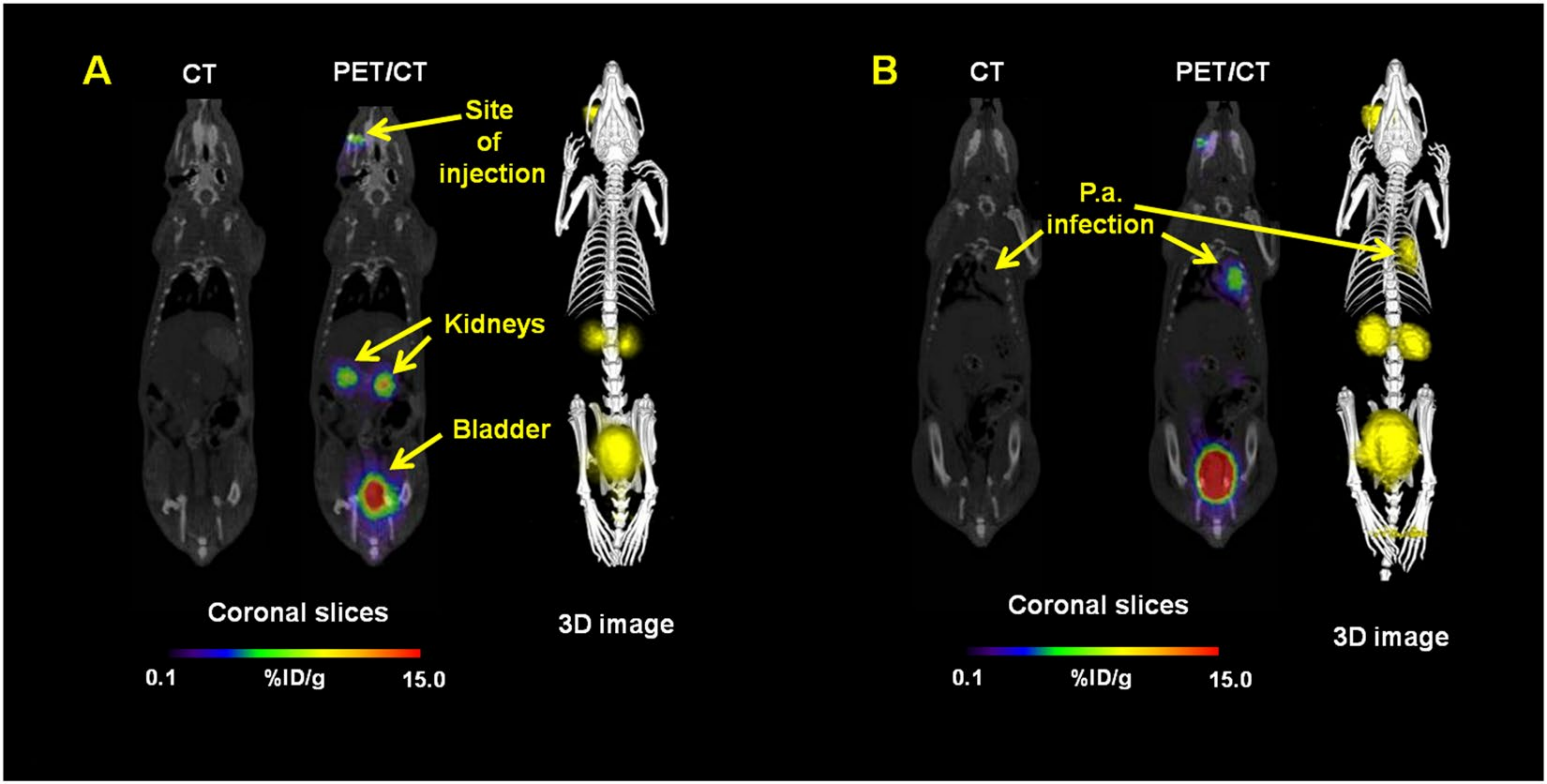
Static PET/CT imaging (coronal slices and 3D volume rendered images) of ^68^Ga-PVD-PAO1 in non-infected (**A**) and intratracheally infected (**B**) Lewis rats 45 min after injection.

**Figure 6:**
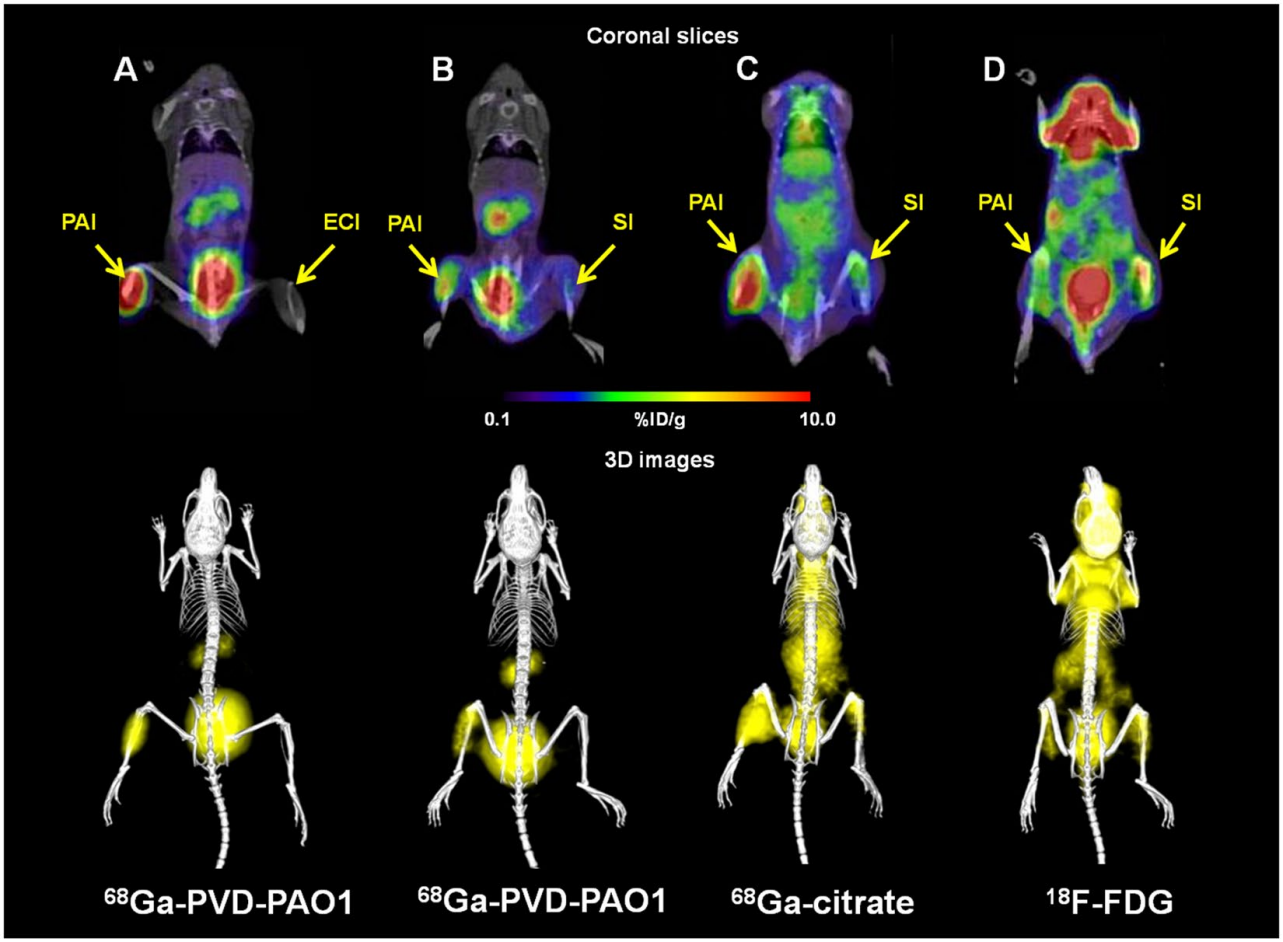
Static PET/CT imaging (coronal slices and 3D volume rendered images) of ^68^Ga-PVD-PAO1 (**A**,**B**) versus ^68^Ga-citrate (**C**) and ^18^F-FDG (**D**) in Balb/c mice muscle infection model 45 min post injection. PAI = *Pseudomonas aeruginosa* infection, ECI = *Escherichia coli* infection and SI = sterile inflammation.

**Figure 7:**
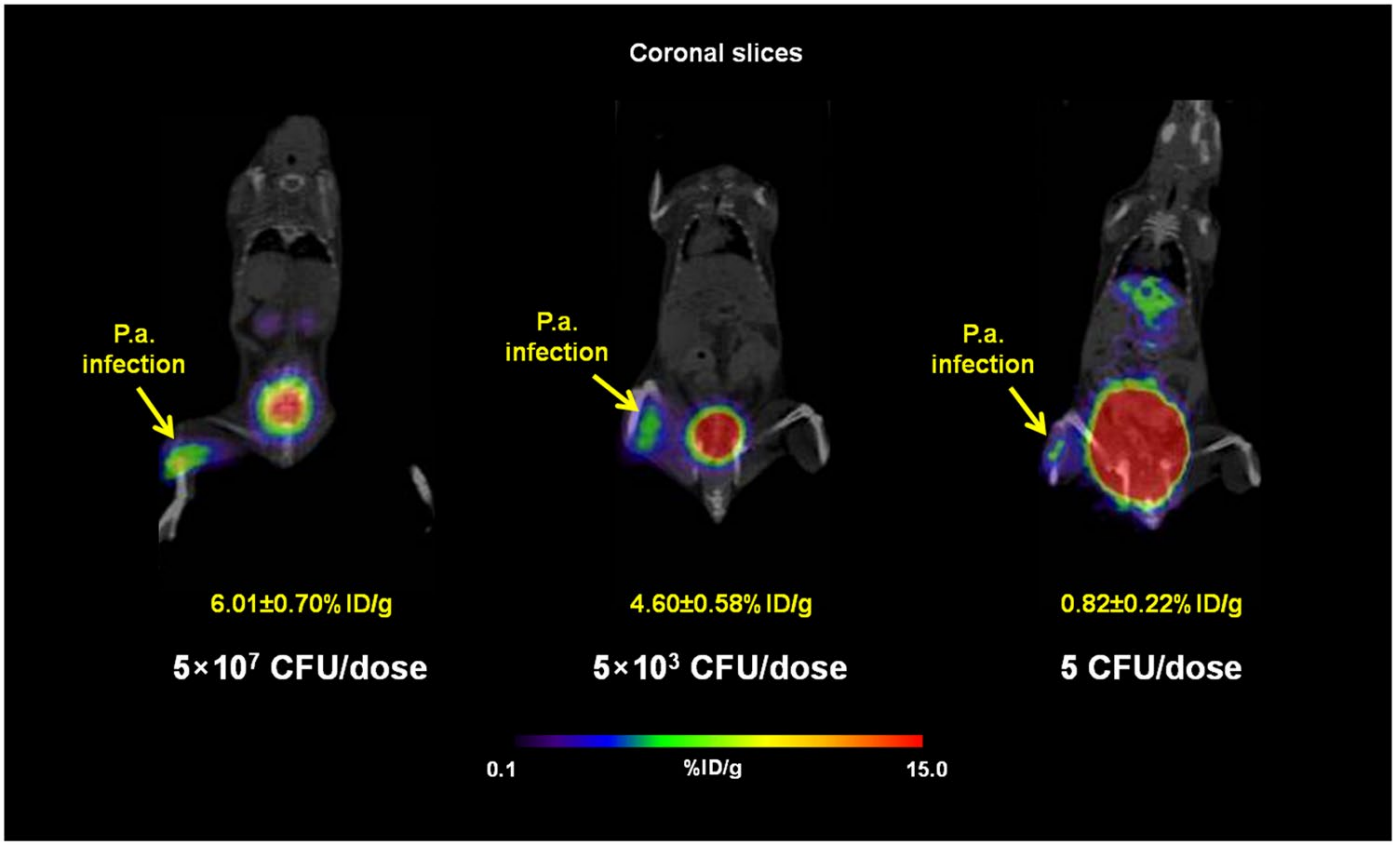
Static PET/CT imaging (coronal slices) of ^68^Ga-PVD-PAO1 in Balb/c mice muscle infection model with different level of infection load 45 min post injection. Standard uptake values (SUV) in *P. aeruginosa* (*P.a*.) infected muscles were calculated by PMOD software (PMOD Technologies Ltd., Zurich, Switzerland) and were 6.01 ± 0.70%ID/g for 5 × 10^7^ CFU/dose; 4.60 ± 0.58%ID/g for 5 × 10^3^ CFU/dose and 0.82 ± 0.22%ID/g for 5 CFU/dose.

MicroPET/CT imaging of Balb/c mice injected with ^68^Ga-PVD-PAO1 confirmed the data from *ex vivo* biodistribution studies. ^68^Ga-PVD-PAO1 was rapidly cleared from the bloodstream with the major excretion route via kidneys (Supplementary Fig. S2B). PET/CT imaging in the rat respiratory infection model showed focal accumulation of ^68^Ga-PVD-PAO1 in the lung (Fig. 5B). No uptake in the lung region was detected in non-infected rats in which the only visible organs were the kidneys and bladder (Fig. 5A). *In vivo* specificity of ^68^Ga-PVD-PAO1 for *Pseudomonas* infection was studied in the mouse muscle infection model. PET/CT images summarized on Fig. 6 display specific accumulation of ^68^Ga-PVD-PAO1 in *P. aeruginosa* infection and much better biodistribution compared to radiopharmaceuticals clinically used for infection imaging. ^68^Ga-PVD-PAO1 showed not only great *in vivo* specificity but also high sensitivity enabling to detect *Pseudomonas* infection in the dose of 5 CFU (Fig. 7).

## Discussion

Novel reliable approaches enabling specific, sensitive and early diagnosis as well as rapid monitoring of infectious diseases are constantly searched for also in the field of nuclear medicine imaging^18^. Currently the diagnosis of infection with radiopharmaceuticals is mainly performed with ^99m^Tc- or ^111^In-labelled leukocytes, ^67^Ga/^68^Ga-citrate, ^99m^Tc-diphosphonates and ^18^F-FDG^19^. However, all of these compounds lack the specificity to discriminate among different infectious pathogens and even between infectious and non-infectious inflammation. The limitations of ^18^F-FDG and ^68^Ga-citrate in terms of pharmacokinetics and specificity were demonstrated in Fig. 6. Recently, various attempts were made to develop specific nuclear imaging agent for infection detection including radiolabelled antimicrobial agents (e.g. peptides, antibiotics), monoclonal antibodies, cytokines, nucleoside analogues (e.g. fialuridine), saccharides (e.g. fluorodeoxysorbitol, maltohexaose, maltose, trehalose), oligomers, polyethyleneglycol liposomes, fluorophores and siderophores^18,20–22^. However, none of these probes was translated to routine clinical practice so far. Imaging agent with clinical translation potential is expected to have the following unique characteristics: (i) high binding affinity to target, (ii) high specificity to target, (iii) high sensitivity, (iv) high contrast, (v) high *in vivo* stability, (vi) low immunogenicity and toxicity and (vii) economical and production feasibility^23^. We believe that from the agents currently under investigation, especially siderophores hold the promise to fulfil these criteria.

Siderophores have high affinity to siderophore transporters that are highly upregulated during infection and are not present in human cells^13,24^. The energy-dependent active uptake of siderophores leads to their accumulation in the target tissue^16^. The low molecular mass of siderophores and their high hydrophilicity ensure rapid diffusion from the circulation into infected tissues, but also rapid clearance from non-target tissue and elimination via renal excretion^16,17^. Radiolabelling of siderophores can be achieved easily by replacing Fe^3+^ in the siderophore by a suitable radionuclide^15,25^. There is no isotope of iron with suitable properties for imaging in terms of half-life and photon emission, however, Ga^3+^ is an isosteric diamagnetic substitute for Fe^3+^ and has been used extensively to characterize siderophore complexes^15,22^. Recently, interest in the isotope Ga-68, a positron emitter, has increased tremendously with the establishment of PET as a clinical imaging modality. Ga-68 can be obtained from a ^68^Ge/^68^Ga generator without the requirement of cyclotron installations and with a half-life of 68 minutes exhibits a very low radiation burden to the patient, typically about half of that of F-18, the most widely used isotope for PET. Moreover, some siderophores can also be labelled with another cyclotron produced positron emitter Zr-89 (t_1/2_ = 78.4 h), which could allow long-term follow-up of infectious process^25^. Typical example of siderophore that can bind Zr-89 is desferrioxamine (DFO). DFO was approved for medical use in the US in 1968 and was clinically used to counteract iron and aluminium overload under the brand name Desferal^26^. Currently, DFO is the only bifunctional chelator used in the clinic for the Zr-89 labelling of monoclonal antibodies^27^.

Radiolabelled siderophores have been investigated in radiopharmaceutical research already in early 1980s^28,29^ and recently were successfully used for the detection of *Staphylococcus aureus* infection^22^ and for preclinical imaging of Invasive Aspergillosis (IA) caused by the fungal pathogen *Aspergillus fumigatus*^16,17^. Triacetylfusarinine C (TAFC), siderophore produced by *A. fumigatus*, was labelled with Ga-68 with high affinity and stability. ^68^Ga-TAFC showed high metabolic stability, favourable pharmacokinetics with rapid renal excretion and high specific uptake in *A. fumigatus* cultures^16,17,30^. Promising properties of ^68^Ga-TAFC were confirmed in imaging studies in a rat IA model that showed high focal uptake in infected lung tissue corresponding to pathological findings seen on CT. These encouraging results have led us to undergo the concept of radiolabelled siderophores for specific infection imaging to test with other pathogens.

In this study we evaluated the potential of radiolabelled *Pseudomonas* produced siderophore, pyoverdine PAO1, as specific agent for bacterial pathogen *P. aeruginosa* infection imaging. The basic mechanism of pyoverdine uptake by *P. aeruginosa* was described in detail in various reviews^31–34^. The ferric-pyoverdine is specifically recognized and subsequently transported by FpVa, specific outer membrane Siderophore Transporters (SITs, also called siderophore receptors) for iron utilization. The energy for this process is provided by the TonB protein, which can span the entire periplasm and functions in coordination with inner membrane proteins ExbB and ExbD during energy transduction. After transport into the periplasm the ferric-pyoverdine is bound by a binding protein that delivers its cargo via the cognate ATP-binding cassette (ABC) transporter into the cytoplasm, where the iron is removed from the complex. Here we attempted to investigate if the Ga-68 labelled pyoverdine can behave similarly to ferric-pyoverdine complex in *P. aeruginosa*.

^68^Ga-PVD-PAO1 showed hydrophilic properties, low protein binding and high stability in human serum. *In vitro* assays displayed rapid and high uptake increasing over time (up to 90 min) by *P. aeruginosa* under iron-deficient conditions, which could be blocked with excess of Fe-PVD-PAO1 or sodium azide. The lower extent of uptake reduction under the addition of sodium azide confirmed the phenomenon that especially in iron-deficient conditions not only transporters are up-regulated, but also siderophore binding proteins, leading to increased cell surface binding of Fe/^68^Ga-siderophores^15^.

It is known that numerous microorganisms possess specific uptake systems not only for native siderophores, but also for siderophores synthesized exclusively by other pathogens, so called “xenosiderophores”^35^. Therefore, *in vitro* uptake studies of selected ^68^Ga-siderophores in *P. aeruginosa* and ^68^Ga-PVD-PAO1 in different microorganisms were performed. None of tested ^68^Ga-siderophores was significantly taken up by *P. aeruginosa* and the uptake of ^68^Ga-PVD-PAO1 was highly specific for *P. aeruginosa* cultures. In animals, ^68^Ga-PVD-PAO1 showed excellent pharmacokinetic properties with rapid renal elimination from non target tissue, selective accumulation in infected tissues and great sensitivity enabling the detection of only five viable cells of *P. aeruginosa*. Moreover, ^68^Ga-PVD-PAO1 displayed better specificity and pharmacokinetic properties than other, clinically used radiopharmaceuticals.

## Materials and Methods

### Chemicals

All reagents were purchased from commercial sources as analytical grade and used without further purification. Pyoverdine PAO1 (PVD-PAO1) isolated from *P. aeruginosa* ATCC 15692 and other siderophores in the study were purchased from EMC Microcollections GmbH (Tuebingen, Germany). ^68^GaCl_3_ was eluted from a ^68^Ge/^68^Ga generator (Eckert & Ziegler Eurotope GmbH, Berlin, Germany) with 0.1 N HCl using the fractionated elution approach. ^18^F-FDG was obtained from the Institute of Nuclear Research (UJV Rez, Prague, Czech Republic).

### Radiolabelling

Radiolabelling of PVD-PAO1 with Ga-68 was performed as follows: 20 μg of PVD-PAO1 dissolved in water (1 μg/μl) were mixed with 30 μl of sodium acetate (155 mg/ml in water) and 300 μl of generator eluate (10–100 MBq of ^68^GaCl_3_). The reaction mixture (pH 3–4) was incubated at 80 °C for l5 min. After the reaction, 100 μl of sodium acetate was added to increase the pH to 5–6. Radiochemical purity (RCP) of ^68^Ga-PVD-PAO1 was analyzed by reversed-phase high-performance liquid chromatography (RP-HPLC) or using instant thin-layer chromatography on silica gel impregnated glass fibres (ITLC-SG).

Siderophores for *in vitro* uptake study in *P. aeruginosa* cultures were radiolabelled with Ga-68 and analyzed as described previously^15,25^, using similar conditions as for PVD-PAO1 labelling. The preparation of ^68^Ga-citrate for *in vivo* specificity challenge was reported elsewhere^36^. Briefly, 300 µl of ^68^Ge/^68^Ga generator eluate were mixed with 80 µl of 0.5 M sodium citrate (pH ∼ 5). The reaction mixture was incubated for 15 min at room temperature.

### Quality control

For determination of radiochemical purity of radiolabelled pyoverdine PAO1, a RP-HPLC gradient method was applied. Dionex UltiMate 3000 (Thermo Scientific, Waltham, MA, USA) and GABI Star (Raytest, Straubenhardt, Germany) radiometric detector were used for RP-HPLC analysis of ^68^Ga-PVD-PAO1. A Nucleosil 120–5 C18 250 × 40 mm column (WATREX, Prague, Czech Republic) with 1 ml/min flow rate was used with the following gradient: acetonitrile (ACN)/0.1% trifluoroacetic acid (TFA)/H_2_O: 0–2 min, 0% ACN; 2–15 min, 0–36% ACN; 15–18 min, 36–60% ACN; 18–19.5 min, 60% ACN; 19.5-20 min, 60-0% ACN; 20–24 min, 0% ACN. ITLC-SG (Varian, Lake Forest, CA, USA) using 1 M ammonium acetate/methanol (1:1) as a mobile phase was used for rapid estimation of the product quality. The retention factor (Rf) of ^68^Ga-PVD-PAO1 was 0.8–1 and Rf of ^68^Ga-eluate was 0.

### *In vitro* characterization

The stability of ^68^Ga-PVD-PAO1 was tested in human serum, 6 mM diethylenetriaminepentaacetic acid (DTPA) and 0.1 M FeCl_3_ solution for 30, 60, and 120 min, respectively, at 37 °C. After incubation, human serum samples were precipitated with acetonitrile and centrifuged (3 min, 2000 g). The supernatant was analyzed by RP-HPLC and ITLC-SG. Samples containing DTPA and FeCl_3_ were analyzed directly.

Protein binding studies were performed by incubating radiolabelled pyoverdine PAO1 in human serum and in phosphate buffered saline (PBS) as a control at 37 °C up to 120 min. After incubation, 25 μl of the sample was separated by size-exclusion chromatography (MicroSpin™ G-50 Columns; Sephadex G-50 (GE Healthcare, Buckinghamshire, UK)) by centrifugation at 2000 g for 2 min. Protein binding of ^68^Ga-PVD-PAO1 was determined by measuring the activity distributed between the column (non-protein-bound) and the eluate (protein-bound) using a γ-counter (2480 Wizard^2^ automatic gamma counter; PerkinElmer, Waltham, USA).

Partition coefficient (log P) of ^68^Ga-PVD-PAO1 was determined as follows. Radiolabelled pyoverdine A in 0.5 ml phosphate-buffered saline (PBS) pH = 7.4 was added to 0.5 ml octanol and the mixture was vigorously vortexed for 15 min. The aqueous and organic solvents were separated by centrifugation and 50 μl aliquots of both layers were collected and measured in a γ-counter. Log P values were calculated from obtained data (mean of n = 6).

All human samples were derived and processed under general ethical criteria accepted at the University Hospital in Olomouc. The informed consent of human participants was obtained in written for usage of biological sample for research purpose in the future. The experiments with human samples were conducted with the approval of Ethics Committee of the University Hospital and the Faculty of Medicine and Dentistry of Palacky University and in Olomouc, Czech Republic.

### Microbial strains and growth conditions

Microbial strains used in the study are listed in Supplementary Table S2. For *in vitro* uptake experiments, strains were grown in 10 ml of Yeast Extract-Peptone-Glucose (YPG) medium (1% of each peptone and yeast extract and 2% of glucose) in 25 ml Erlenmeyer flasks closed by cotton stoppers. Flasks were shaken at 90 rpm at 30 °C for 16–24 hours to prepare an iron-limited culture. A control iron-replete culture was prepared at the same conditions except for the YPG medium being supplemented with 3 µM FeSO_4_. *Pseudomonas aeruginosa* ATCC 15692 and *Escherichia coli* ATCC 10536 for *in vivo* experiments were cultivated in Petri dishes containing the solid medium Luria-Bertani (LB; 1% tryptone, 0.5% yeast extract, 1% NaCl, 2% agar) for 24 h at 30 °C. Then, the single colony from the Petri dish was transferred to a 500 ml Erlenmeyer flask containing 100 ml of LB broth and incubated on a shaker (200 rpm) at 30 °C. After 24 h, the cell suspension was centrifuged (8000 g, 10 min, 10 °C) and the pellet was diluted with PBS to reach the final number of viability cells of 10^9^ CFU/ml.

### *In vitro* uptake assays in *P. aeruginosa* cultures

For the monitoring of uptake over time, ^68^Ga-PVD-PAO1 was incubated in triplicates with iron-deficient *P. aeruginosa* culture for 10, 20, 30, 45, 60, and 90 min at 37 °C with or without blocking solution (Fe-PVD-PAO1) in Eppendorf tubes. Incubation was interrupted by 5-min centrifugation at 21000 g, supernatant removal and rapid rinsing with ice-cold Tris buffer. The same procedure was repeated twice. After the last centrifugation and supernatant removal Eppendorf tubes containing the microbial sediment were weighed and counted in a γ-counter. Results were expressed as percentage of applied dose per gram of microbial culture (%AD/g).

For the uptake assays, ^68^Ga-PVD-PAO1 was incubated in triplicates with iron-sufficient or iron-deficient *P. aeruginosa* for 45 min at 37 °C with and without sodium azide or excess of Fe-PVD-PAO1. After the incubation samples were treated as described above. *In vitro* uptake of different Ga-68 labelled siderophores (triacetylfusarinine C (TAFC), ornibactin (ORNB), ferrioxamine E (FOXE), ferrichrome A (FCHA) and aerobactin (AERO)) was tested in *P. aeruginosa* under iron-deficient conditions. ^68^Ga-siderophores were incubated in *P. aeruginosa* in triplicates for 45 min at 37 °C. After the incubation samples were treated as described above.

### *In vitro* uptake in various microorganisms

*In vitro* uptake was studied in microbial iron-deficient cultures listed in Supplementary Table S2. Microbial cultures were incubated in Eppendorf tubes in triplicates with ^68^Ga-PVD-PAO1 at 37 °C for 45 min. The uptake was interrupted by 5-min centrifugation at 21000 g. The supernatant was removed and the sediment was disturbed by 1 ml of ice-cold Tris buffer and subsequent whirling. The same procedure was repeated twice. After the last centrifugation and supernatant removal Eppendorf tubes containing the microbial sediment were weighed and counted in a γ-counter. Results were expressed as percentage of applied dose per gram of microbial culture (%AD/g).

### Animal experiments

All animal experiments were conducted in accordance with regulations and guidelines of the Czech Animal Protection Act (No. 246/1992), and with the approval of the Czech Ministry of Education, Youth, and Sports (MSMT-21275/2016-2), and the institutional Animal Welfare Committee of the Faculty of Medicine and Dentistry of Palacky University in Olomouc. The studies were performed using female 8–10-week-old Balb/c mice and female 2–3-months-old Lewis rats (Envigo, Horst, The Netherlands). The number of animals was reduced as much as possible (n = 3 per group and time point) for all *in vivo* experiments. The introduction of bacterial infection into animals, tracer injection as well as small animal imaging was carried out under 2% isoflurane anaesthesia (FORANE, Abbott Laboratories, Abbott Park, IL, USA) to minimize animal suffering and to prevent animal motion.

### *Ex vivo* biodistribution in mice and rats

Non-infected mice as well as *P. aeruginosa* infected and non-infected rats were retro-orbitally (r.o.) injected with ^68^Ga-PVD-PAO1 (1–2 MBq and 0.5–1 µg of pyoverdine PAO1 per mouse or rat). Mice were sacrificed by cervical dislocation 30 and 90 min after injection, while rats were killed by exsanguination 45 min post-injection (p.i.). Organs and tissues of interest (blood, spleen, pancreas, stomach, intestines, kidneys, liver, heart, lung, muscle and femur) were removed and weighed. The amount of radioactivity in the samples was measured in a γ-counter. Results were expressed as percentage of injected dose per gram of organ (%ID/g).

### Animal infection models

*In vivo* uptake of radiolabelled tracers was studied in acute respiratory^37^ and muscle infection^30^ models. Rats were infected intratracheally with *P. aeruginosa* ATCC 15692 under inhalation anaesthesia. *P. aeruginosa* (10^8^ CFU/dose in 100 µl) was administered using TELE PACK VET X LED system equipped with a flexible endoscope (Karl Storz GmbH & Co. KG, Tuttlingen, Germany). Five hours after infection rats underwent *ex vivo* biodistribution or imaging studies. Muscle infection model was established by intramuscular (i.m.) injection of *P. aeruginosa* ATCC 15692 (5 to 5 × 10^7^ CFU/dose in 50 µl) in mice 5 hours before PET/CT imaging. For *in vivo* specificity challenge the infection was induced in the left hind muscle with *P. aeruginosa* (5 × 10^7^ CFU/dose in 50 µl 5 h prior imaging) and in the right hind muscle with *E. coli* ATCC 10536 (5 × 10^7^ CFU/dose in 50 µl 5 h prior imaging) or with turpentine oil (50 µl 24 h prior imaging) in mice.

### Animal imaging studies

MicroPET and CT images were acquired with an Albira PET/SPECT/CT small animal imaging system (Bruker Biospin Corporation, Woodbridge, CT, USA). Mice and rats were r.o. injected with radiolabelled tracer in a dose of 5–10 MBq corresponding to 1–2 μg of pyoverdine PAO1 per animal. Anaesthetized animals were placed in a prone position in the Albira system before the start of imaging. Static PET/CT images were acquired over 40 min starting 30 and 90 min after injection for normal biodistribution studies and 45 min post-injection for infection models imaging. A 10-min PET scan (axial FOV 148 mm) was performed, followed by a triple CT scan (axial FOV 65 mm, 45 kVp, 400 μA, at 400 projections). Scans were reconstructed with the Albira software (Bruker Biospin Corporation, Woodbridge, CT, USA) using the maximum likelihood expectation maximization (MLEM) and filtered backprojection (FBP) algorithms. After reconstruction, acquired data was viewed and analyzed with PMOD software (PMOD Technologies Ltd., Zurich, Switzerland). 3D volume rendered images were obtained using VolView software (Kitware, Clifton Park, NY, USA).

### Statistical analysis

Student’s t-test (level of significance, P < 0.005) was used to determine the significance of differences in the *ex vivo* biodistribution data of infected and non-infected rats. Analysis was performed using Microsoft Office Excel 2007.

## Conclusion

We have shown that ^68^Ga-PVD-PAO1 can be used for the detection of *P. aeruginosa* infection with high specificity and sensitivity. The high and specific uptake of ^68^Ga-PVD-PAO1 by *P. aeruginosa* was confirmed both *in vitro* and *in vivo*, proving the potential of pyoverdine PAO1 for specific imaging of *Pseudomonas* infections. Biodistribution studies in non-infected animals showed excellent *in vivo* behaviour of ^68^Ga-PVD-PAO1 with rapid renal excretion and no accumulation in any organ. Animal infection models displayed focal uptake of ^68^Ga-PVD-PAO1 with high sensitivity in infected tissue and much better pharmacokinetics than clinically used radiopharmaceuticals. Thus, ^68^Ga-PVD-PAO1 seems to be a promising new PET agent for specific *P. aeruginosa* imaging. Moreover, we have proved that the concept of radiolabelled siderophores for specific infection imaging is transferable to different pathogens.

## Electronic supplementary material

Supplementary information

## Data Availability

All data generated or analyzed in this study are included in this published article and its supplementary information files.
