# Supplementary material for: Imaging of Pseudomonas aeruginosa infection with Ga-68 labelled pyoverdine for positron emission tomography
Source: Sci Rep. 2018 Oct 24;8:15698. doi: 10.1038/s41598-018-33895-w (PMC6200719; doi:10.1038/s41598-018-33895-w)
Supplement: Supplementary file 1 — Supplementary information [file 41598_2018_33895_MOESM1_ESM.pdf]

Imaging of *Pseudomonas aeruginosa* infection with Ga-68 labelled pyoverdine for positron emission tomography

Milos Petrik[1], Eva Umlaufova[1], Vladislav Raclavsky[2], Andrea Palyzova[3], Vladimir Havlicek[3,4], Hubertus Haas[5], Zbynek Novy[1], Dalibor Dolezal[1], Marian Hajduch[1] and Clemens Decristoforo[6]

[1]Institute of Molecular and Translational Medicine, Faculty of Medicine and Dentistry, Palacky University, Olomouc, Czech Republic

[2]Department of Microbiology, Faculty of Medicine and Dentistry, Palacky University, Olomouc, Czech Republic

[3]Institute of Microbiology of the Czech Academy of Sciences, v.v.i., Prague, Czech Republic

[4]Regional Centre of Advanced Technologies and Materials, Department of Analytical Chemistry, Faculty of Science, Palacky University, Olomouc, Czech Republic

[5]Division of Molecular Biology/Biocenter, Medical University Innsbruck, Innsbruck, Austria

[6]Clinical Department of Nuclear Medicine, Medical University Innsbruck, Innsbruck, Austria

Table of contents

Supplementary Figure S1: Quality control of ^{68}Ga-PVD-PAO1 using high performance liquid chromatography (A) and instant thin-layer chromatography on silica gel impregnated glass fiber sheets (B).

Supplementary Figure S2: (A) Biodistribution of ^{68}Ga-PVD-PAO1 in non-infected Balb/c mice 30 a 90 min after injection. (B) Static PET/CT imaging (3D volume rendered images) of ^{68}Ga-PVD-PAO1 in non-infected Balb/c mice 30 and 90 min after injection.

Supplementary Table S1: *In vitro* characterization of ^{68}Ga-PVD-PAO1. Log P, protein binding (expressed as % of protein bound activity of the total activity used) and stability in human serum, 0.1 M $FeCl_3$ and 6 mM DTPA.

Supplementary Table S2. Microbial strains used in the study.

Supplementary Figure S1: Quality control of ^{68}Ga-PVD-PAO1 using high performance liquid chromatography (A) and instant thin-layer chromatography on silica gel impregnated glass fiber sheets (B).

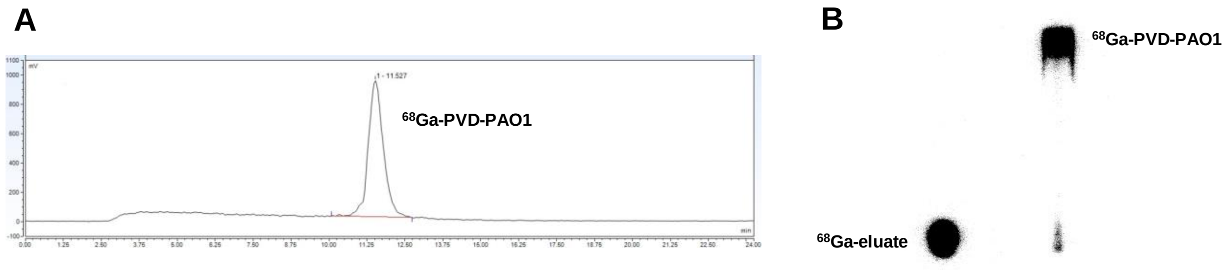

Supplementary Figure S2: (A) Biodistribution of ^{68}Ga-PVD-PAO1 in non-infected Balb/c mice 30 a 90 min after injection. (B) Static PET/CT imaging (3D volume rendered images) of ^{68}Ga-PVD-PAO1 in non-infected Balb/c mice 30 and 90 min after injection.

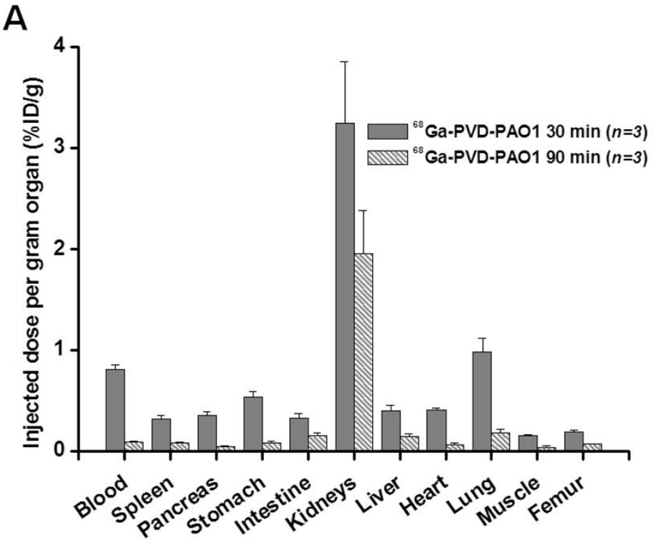

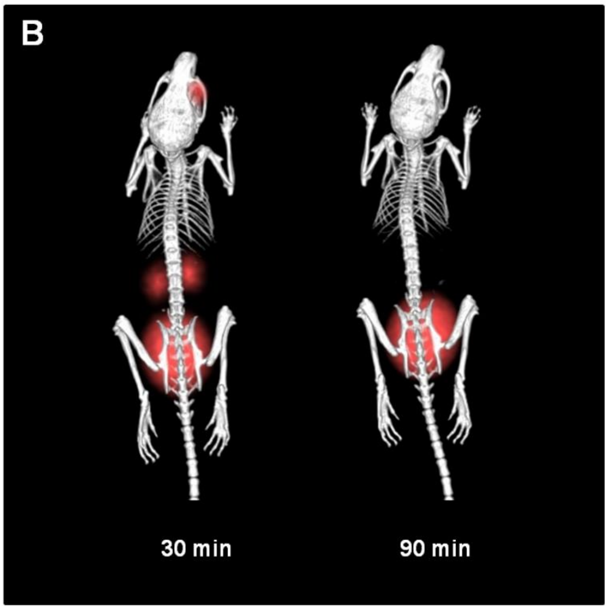

Supplementary Table S1: *In vitro* characterization of ^{68}Ga-PVD-PAO1. Log P, protein binding (expressed as % of protein bound activity of the total activity used) and stability in human serum, 0.1 M $FeCl_3$ and 6 mM DTPA.

**^{68}Ga-siderophore**	**Log P (n=6)**	**Incubation time (min)**	**Protein binding (%) (n=3)**	**Stability in human serum (%) (n=3)**	**Stability in iron solution (%) (n=3)**	**Stability in DTPA solution (%) (n=3)**
**^{68}Ga-PVD-PAO1**	-3.07±0.08	30	2.31±0.93	97.9±0.52	0.53±0.32	94.8±2.21
		60	2.79±1.14	96.6±1.19	0.37±0.21	95.7±0.62
		120	2.46±0.73	95.6±2.34	0.17±0.09	89.7±2.73

Supplementary Table S2. Microbial strains used in the study.

*No refers to the Collection of clinical isolates of the Department of Microbiology, Faculty of Medicine & Dentistry, Palacky University Olomouc.

CF – cystic fibrosis; COPD – chronic obstructive pulmonary disease; CRC – colorectal cancer

Species	Isolate No*	Origin
Acinetobacter baumannii	2377	sputum of a CF patient
Burkholderia cenocepacia	2029	sputum of a CF patient, ST-32 strain
Burkholderia multivorans	1926	bronchial aspirate from an acute respiratory failure patient
Candida albicans	1265	sputum of a CF patient
Escherichia coli	ATCC 10536	ATCC
Escherichia coli	2463	rectal swab from a patient suffering from haemorrhoids
Klebsiella pneumonia	2102	rectal swab from a CRC patient
Listeria monocytogenes	---	QC control strain from national authority
Pseudomonas aeruginosa	ATCC 15692	ATCC
Pseudomonas aeruginosa	1019	sputum of a COPD patient
Pseudomonas grimontii	754	sputum of a COPD patient
Pseudomonas monteilii	2260	rectal swab from a low birth-weight newborn
Staphylococcus aureus	230	sputum of a CF patient
Stenotrophomonas maltophilia	303	sputum of a CF patient
Streptococcus agalactiae	487	sputum of a CF patient
Yersinia enterocolitica	2871	rectal swab from a patient suffering from gallstones
